# Acid Mine Drainage Precipitates from Mining Effluents as Adsorbents of Organic Pollutants for Water Treatment

**DOI:** 10.3390/molecules29153521

**Published:** 2024-07-26

**Authors:** Marta S. F. Oliveira, Ouissal Assila, António M. Fonseca, Pier Parpot, Teresa Valente, Elisabetta Rombi, Isabel C. Neves

**Affiliations:** 1 CQUM, Chemistry Department, Centre of Chemistry, Campus de Gualtar, University of Minho, 4710-057 Braga, Portugal; martaoliveira@quimica.uminho.pt (M.S.F.O.); ouissal.assila@usmba.ac.ma (O.A.); amcf@quimica.uminho.pt (A.M.F.); parpot@quimica.uminho.pt (P.P.); 2 CEB, Centre of Biological Engineering, Campus de Gualtar, University of Minho, 4710-057 Braga, Portugal; 3 ICT, Institute of Earth Sciences, Pole of the University of Minho, 4710-057 Braga, Portugal; teresav@dct.uminho.pt; 4 Department of Chemical and Geological Sciences, University of Cagliari, 09042 Monserrato, Italy; rombi@unica.it

**Keywords:** acid mine drainage (AMD), adsorption, dyes, Box–Behnken Design, water treatment, circular economy

## Abstract

Acid mine drainage (AMD) is one of the main environmental problems associated with mining activity, whether the mine is operational or abandoned. In this work, several precipitates from this mine drainage generated by the oxidation of sulfide minerals, when exposed to weathering, were used as adsorbents. Such AMD precipitates from abandoned Portuguese mines (AGO, AGO-1, CF, and V9) were compared with two raw materials from Morocco (ClayMA and pyrophyllite) in terms of their efficiency in wastewater treatment. Different analytical techniques, such as XRD diffraction (XRD), Fourier Transform Infrared spectroscopy (FTIR), N_2_ adsorption isotherms, and Scanning Electron Microscopy (SEM) with Energy Dispersive X-ray (EDX) were used to characterize these natural materials. The adsorption properties were studied by optimizing different experimental factors, such as type of adsorbent, adsorbent mass, and dye concentration by the Box–Behnken Design model, using methylene blue (MB) and crystal violet (CV) compounds as organic pollutants. The obtained kinetic data were examined using the pseudo-first and pseudo-second order equations, and the equilibrium adsorption data were studied using the Freundlich and Langmuir models. The adsorption behavior of the different adsorbents was perfectly fitted by the pseudo-second order kinetic model and the Langmuir isotherm. The most efficient adsorbent for both dyes was AGO-1 due to the presence of the cellulose molecules, with q_m_ equal to 40.5 and 16.0 mg/g for CV and MB, respectively. This study confirms the possibility of employing AMD precipitates to adsorb organic pollutants in water, providing valuable information for developing future affordable solutions to reduce the wastes associated with mining activity.

## 1. Introduction

Various technologies have been developed to remove inorganic and organic pollutants from wastewater, including chemical precipitation, oxidation, electrolysis, reverse osmosis, membrane separation, biofilm technology, ion exchange, photocatalysis, and adsorption. Among these technologies, adsorption processes are preferred for treating wastewater due to their simplicity of operation and low cost [1,2]. The use of solid wastes as adsorbents for removing organic or inorganic pollutants from wastewater contributes to minimizing, recovering, and reusing waste, allowing the development of increasingly sustainable processes within the circular economy. Many waste products have been transformed into low-cost adsorbents and used for water treatment, such as agricultural wastes, industrial wastes, soil, and ore materials [1,2,3].

Among ore materials, precipitates from natural or induced purification processes of mining effluents, such as acid mine drainage (AMD), have attracted interest in being used as adsorbents in the adsorption process for water treatment [4,5,6]. AMD is a primary environmental concern linked to mining activity whether the mine is operational or abandoned. This is attributed to the elevated levels of toxic element concentrations and the low pH (often <3), which facilitate the dissolution of specific minerals upon exposure to such an acidic environment [7,8,9,10]. This is the case for sulfides, of which pyrite (FeS_2_) is a common example, which undergo weathering through processes of biogeochemical nature, developing high acidity and electrical conductivity and a high concentration of sulfate and other elements considered potentially toxic such as cadmium (Cd), zinc (Zn), lead (Pb), among others [9]. These pollutants trigger phenomena that manifest in the contamination of the surface and underground water, soil, and sediments and often promote the degradation of the entire ecosystem. The environmental impact caused by AMD affects the source area where ore deposits are exploited and may affect more distant regions through dispersion processes due to wind and water [11,12,13]. In addition, large amounts of ochre precipitates are formed due to natural dilution and neutralization processes in AMD-affected watercourses.

Conventional passive treatment systems are usually constructed in abandoned mining areas to minimize the AMD consequences. Limestone and other alkaline materials are often applied to promote neutralization [10,14]. The resulting sludge, which constitutes waste that must be given an appropriate final destination, is generally composed of large amounts of iron, mainly in the form of iron oxides-hydroxides of low crystallinity [4,15]. In this light, reusing this AMD precipitate as an adsorbent may be the key element for a more sustainable water treatment process, and a key for a circular economy [16,17].

In this work, wastes in the form of AMD precipitates associated with mining activity in Portugal (AGO, AGO-1, CF, and V9) were used as adsorbents and compared with materials available in nature from Morocco (natural clay and Pyrophyllite), in the adsorption of dyes from aqueous solutions. These adsorbents were found to have a high removal capacity for dyes with localized positive charges. Methylene blue (MB) and crystal violet (CV) dyes were investigated as the organic pollutants. These dyes are currently used as drugs or additives in the industry [18,19]. MB, also known as methylthioninium chloride, is used as a medicament in treating vasodilator shock during and after surgery requiring cardiopulmonary bypass [18]. CV, formerly known as methyl violet 10B, is used as a histological stain, particularly in Gram staining for classifying bacteria, and is used as a topical antiseptic. In addition, it is used in textiles, paper dye, or ink for printing, ball-point pens, and inkjet printers [19]. Both dyes present a positive charge in their structure, which favors the adsorption by materials with negative charges in their frameworks. CV has a higher molecular weight, and it is bulkier than MB in terms of molecular structure (Figure S1).

The adsorption process was optimized using the Box–Behnken Design (BBD) as an experimental model for response surface methodology (RSM). In mathematical and statistical techniques, RSM is currently used for fitting a quadratic model, which describes the relationship between independent factors and their interaction effects to determine the optimum values for input parameters in all processes. BBD offers three-level factors and their interaction terms for a comprehensive experimental model using economically feasible experiments [20,21].

For both the investigated dyes, kinetic studies on the different adsorbents showed that they follow the pseudo-second order model, while equilibrium data were fitted with the Langmuir model. The most effective adsorbent in removing methylene blue was pyrophyllite (q_m_ = 30.8 mg/g), whereas in the case of crystal violet it was AGO-1 (q_m_ = 40.5 mg/g).

## 2. Results and Discussion

### 2.1. Characterization of the Adsorbents

**Figure 1:**
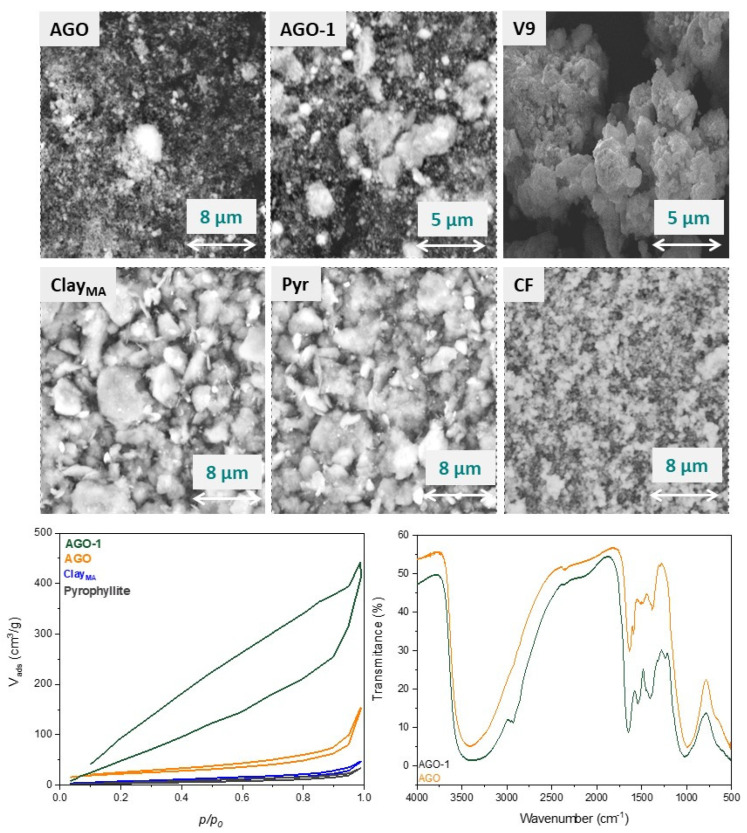
SEM images of the AMD precipitates and raw materials; N_2_ adsorption/desorption isotherms of AGO-1, AGO, Clay_MA_, and pyrophyllite; and FTIR spectra of the AGO-1 and AGO adsorbents.

Figure 1 displays the results of several techniques used to characterize the materials selected for this work. From SEM analysis, AMD precipitates present smaller aggregates compared to the raw samples, as expected. Clay_MA_ and pyrophyllite (Pyr) from Morocco are heterogeneous in both particle size and shape, forming voids between the aggregates.

AMD precipitates show differences in their morphologies. For AGO and AGO-1, the presence of rounded particles of very different sizes can be observed, whose number is remarkably higher in the case of AGO-1, probably due to the presence of organic matter such as algae. Conversely, V9, the sludge resulting from natural attenuation, and CF, the sludge derived from the passive treatment of mining effluents, exhibit the existence of uniformly sized small, rounded particles.

The results of EDX analysis are reported in Table 1, where, for Clay_MA_, the chemical composition determined by Inductively Coupled Plasma-Optical Emission Spectroscopy (ICP-OES) is also shown [22]. The typical elements expected for this material were detected for all the samples. Besides oxygen, which is present in high amounts in all the solids investigated, C and Fe are the main components in AMD precipitates. In contrast, high amounts of Si and Al are present in both Clay_MA_ and Pyr, the former also containing a significant amount of Fe. Compared to AGO, the increase in the wt% of oxygen and iron observed for AGO-1 is probably related to the presence of algae. The differences in ADM precipitates are related to the source and geological background where these samples were collected.

Figure 1 shows the N_2_ adsorption/desorption isotherms of selected precipitates and of the raw materials from Morocco, and their textural properties are summarized in Table S1. All the isotherms are typical for these materials [23,24] with hysteresis loops in the 0.20–1.0 range of *p*/*p*_0_; they can be defined as type IV isotherms according to the IUPAC classification. In the case of AGO-1, a much larger hysteresis loop is observable; it also shows the greatest values of both specific surface area (S_BET_ = 369 m^2^/g) and total pore volume (Vp = 0.489 cm^3^/g), which are significantly higher than those of the AGO sample (S_BET_ = 79 m^2^/g and Vp = 0.124 cm^3^/g). The difference in the values may be ascribed to the presence of the organic matter coming from the algae. The increase could be due to the decomposition of this matter during some treatments that eliminate it, leaving void spaces. It can be noted from Table S1 that Pyr and Clay_MA_ present considerably lower values of S_BET_ (12 and 23 m^2^/g, respectively) and pore volumes an order of magnitude smaller than those of the precipitates.

The presence of the algae filaments was investigated by FTIR analysis (Figure 1). The AMD precipitates present similar FTIR spectra, with a broad band at 3400 cm^−1^ ascribable to ν(OH) of hydroxyl groups characteristic of oxyhydroxide minerals with iron and water [25]. The band observed at 1630 cm^−1^ is instead associated with the H–O–H vibrations of free water molecules [25]. The presence of organic matter in the spectrum of AGO-1 is confirmed by the appearance of the ν(CH stretching) and ν(CH bending) bands around 2900 cm^−1^ and 1400 cm^−1^, respectively. The band at 1020 cm^−1^ is associated with the asymmetric vibration of C–O–C from cellulose molecules [26,27] (Figure S2).

These results support the hypothesis that the different elemental compositions between AGO and AGO-1 (Table 1) are due to the presence of organic matter from the algae filaments in the latter sample. This is likely because these precipitates are in contact with soil and water, essential sources of organic matter. Indeed, these AMD precipitates exhibit an amorphous or very low crystallinity character, as indicated by the presence of broader peaks rather than well-defined ones in the XRD analysis, which identified ferrihydrite iron hydroxide at d-values of 2.5 Å and 1.5 Å (Figure S3).

### 2.2. Optimization of the Adsorption Process

For the optimization of the adsorption process by the Box–Behnken Design (BBD) model, three adsorbents, one from AMD (AGO) and two raw materials from Morocco (Clay_MA_ and Pyr), were selected. The BBD of the RSM was employed to determine the individual and combined effects of three variables, viz. type of adsorbent, concentration of dye, and adsorbent mass, and optimize the parameters of the adsorption process (Table S2). Three response surface regression models were developed to estimate the adsorption efficacy of the targeted dyes from the adsorbent materials. Table S3 summarizes the experimental data for the removal of MB or CV by the chosen adsorbents, AGO, phyrophillite, and Clay_MA_ as in function of the responses Y_1_ (removal efficiency R, %) and Y_2_ (q_ads_, mg/g).

ANOVA statistically assessed the quality of the models for both responses, Y_1_ and Y_2_ (Tables S4 and S5, respectively). The calculated *p*-values in Tables S4 and S5 for both dyes were <0.05, which indicated that the applied models are statistically significant, and values greater than 0.1000 indicated the model terms are not significant [20,21,22]. The second-order polynomial equations (Equations (S1)–(S4) in Table S6) for both responses were also derived from these models for the MB and CV dyes, along with their individual parameters and interaction conditions. In the equations presented in Table S6, the positive or negative signs of the coefficients signify the synergistic or antagonistic effects on the responses, respectively [21,22].

In the second-order polynomial equations, X_1_, X_2_, and X_3_ correspond to the independent variables type of adsorbent, mass of adsorbent, and dye concentration, respectively. Concerning the removal efficiency (Y_1_, Equations (S1) and (S3) in Table S6), X_1_ and X_2_ are always significant, whereas X_2_X_3_ and X_1_X_2_ interactions are significant for MB and CV, respectively. As for the Y_2_ response (q_ads_, Equations (S2) and (S4) in Table S6), all three independent variables are significant model terms for both dyes. The properties of the adsorbents and their mass are important variables for the adsorption of the dyes. For both dyes, correlation coefficients (R^2^) for response Y_2_ were higher than for Y_1_ (Table 2), being 98.44% and 98.10% for MB and CV, respectively, which demonstrates that the application of developed models is well fitted to the experimental data and is highly predictive [20,21,22].

**Figure 2:**
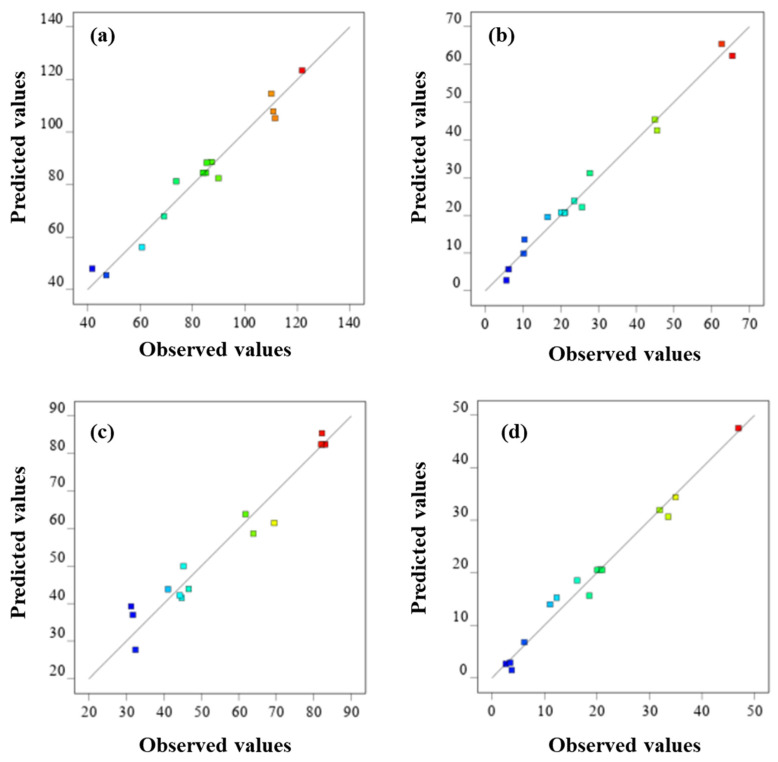
Predicted values vs. actual values for the Y_1_ and Y_2_ responses: (**a**,**b**) MB and (**c**,**d**) CV adsorption.

Figure 2 shows the highly positive correlation between the predicted and the experimental values for MB and CV adsorption as a function of the Y_1_ and Y_2_ responses. In all cases, the data points on the normal probability plot of the residuals lie practically close to a straight line, indicating that the models are suitable and perform well for both MB and CV responses.

**Figure 3:**
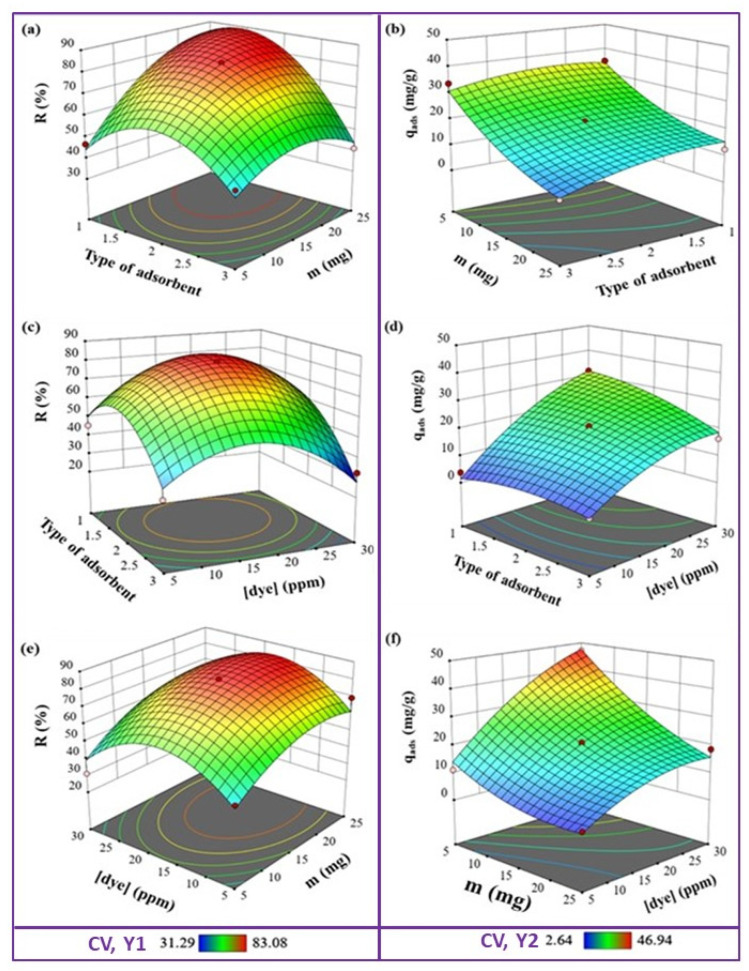
3D-RSM plots for CV adsorption, where the response is reported as a function of two of the independent variables while keeping the third constant: Y_1_ (**a**,**c**,**e**) and Y_2_ (**b**,**d**,**f**).

Three-dimensional responses (3-D RSM) were obtained to help analyze of the quadratic models presented in Table 2. Figures S4 and Figure 3 display the 3-D RSM curves of removal efficiency (%) and uptake (mg/g) for MB and CV, respectively.

It can be observed that both responses increase as the adsorbent mass is enhanced from 5 to 25 mg, regardless of the type of adsorbent. In Figure S4c,d, the combined binary effect of adsorbent type (X_1_) and dye concentration (X_3_) shows that, whatever the adsorbent, the removal efficiency is lower when increasing the dye concentration, whereas the adsorbed quantity is higher. Figure S4e,f show the combined binary effect of the adsorbent mass (X_2_) and dye concentration (X_3_), displaying that, at a fixed mass value, removal efficiency and uptake increase while decreasing the dye concentration, suggesting that the X_2_X_3_ interaction is significant enough to maximize MB adsorption.

Likewise, for the adsorption of CV, the binary effect of the adsorbent mass (X_2_) and type (X_1_) on both responses, Y_1_ and Y_2_, is shown in Figure 3a,b, respectively. The maximum removal capacity was observed for Clay_MA_ with 15 mg of adsorbent. In Figure 3c,d, the combined effect of the adsorbent type (X_1_) and dye concentration (X_3_) seems to affect both responses similarly, which confirms that these terms are significant.

Concerning the removal efficiency, for a certain fixed mass, it is higher when using pyrophyllite and dye concentrations between 15 and 20 ppm. In addition, Figure 3e,f show the binary effect of the adsorbent mass (X_2_) and dye concentration (X_3_), highlighting the importance of the X_2_ parameter, whose 15 mg value allowed the highest removal efficiency (Figure 3e). As for the uptake (Figure 3f), the highest amount of adsorbed CV is obtained when a lower adsorbent mass and higher dye concentrations are used.

From these results, Table 3 presents the optimal experimental conditions obtained by the Box–Behnken Design for the adsorption of MB and CV, considering the best uptake results.

**Figure 4:**
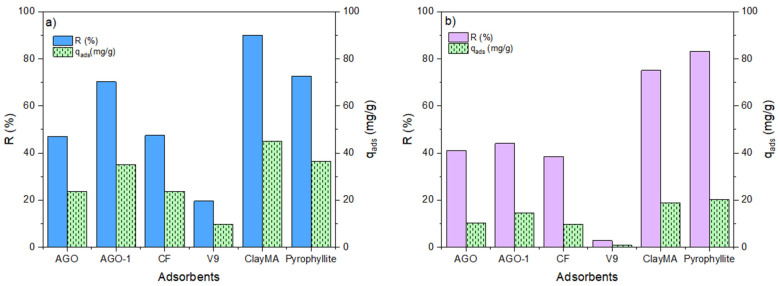
Comparison of the removal efficiency (R, %) and uptake (q_ads_, mg/g) of the investigated materials in the adsorption of MB (**a**) and CV (**b**).

The adequacy of the proposed mathematical model for the adsorption of MB and CV on the different adsorbents was evaluated using the optimal conditions (30 ppm of MB and 17.5 ppm of CV, with 15 mg of each adsorbent), to compare AMD precipitates with raw materials. Figure 4 presents the results, in terms of removal efficiency and uptake, obtained for the adsorption of MB (Figure 4a) and CV (Figure 4b) on all the investigated materials.

As expected, the raw materials from Morocco present a higher removal efficiency for both dyes, with Clay_MA_ showing a better performance in the adsorption of MB, and pyrophyllite in that of CV. However, the uptake (q_ads_, mg/g) of MB is higher than that of CV on both the raw adsorbents. The enhanced behavior of the raw adsorbents can be reasonably due to the presence of a higher Al content (Table 1) that induces a higher negative charge in their frameworks despite presenting lower S_BET_ and Vp compared to AGO. In the case of AMD precipitates, the MB removal efficiency is 70.2% for AGO-1, followed by similar values for AGO and CF (47.1 and 47.5%, respectively) and then by the worst result (ca. 20%) for V9 (Figure 4a). A similar trend can be observed for the uptake, whose values are in the following order: AGO-1 > CF ≈ AGO > V9 (Figure 4a). In the case of CV, due to the molecule’s properties (Figure S1), the adsorption performances are lower than for MB, regardless of the adsorbent used. Noteworthy, AGO-1 still shows the best results, confirming that the adsorption capacities of AGO materials can be improved by the presence of algae, due to the resulting increase in both S_BET_ and Vp. The removal efficiencies (Figure 4b) are in the following order: AGO-1 (44.0%) > AGO (41.1%) > CF (38.5%) > V9 (2.8%), and the CV uptake shows the same trend.

### 2.3. Adsorption Studies

For adsorption kinetics and equilibrium studies, AGO-1 was used to represent the AMD precipitates and compare them with Clay_MA_ and pyrophyllite. Two kinetic models were examined to fit the experimental data, namely the pseudo-first order (PFO) and the pseudo-second order (PSO) [28]. PFO assumes that the adsorbed amount is proportional to the number of vacant adsorption sites, whereas PSO assumes that the adsorbed amount is proportional to the square of the number of vacant adsorption sites and is related to chemisorption [28]. The models are expressed in linear form by Equations (1) and (2) for PFO and PSO, respectively:

(1) $$\mathrm{Ln}\left[q_{e}-q(t)\right]=\mathrm{Ln}\left(q_{e}\right)-K_{1}t$$

where *K*_1_ is the constant rate of Lagergren’s first-order (min^−1^), qt and qe are the amounts of the adsorbed dyes by the adsorbents at time t and equilibrium (mg.g^−1^), respectively, and t is the contact time (min).

(2) $$\left(\frac{t}{q_{t}}\right)=(\frac{1}{K_{2}q_{e}^{2}})-(\frac{1}{q_{e}})t$$

where *K*_2_ is the constant rate of Ho’s second-order (mg.g^−1^.min^−1^), and the other parameters are the same as already defined in the previous equation. The kinetic equations for the three investigated adsorbents are shown in Table S7.

Compared to the raw adsorbents, AGO-1 shows the highest values of R^2^ by using the PSO model (0.9916 for MB and 0.9987 for CV) and the lowest ones (0.4088 for MB and 0.2743 for CV) by fitting the data with PFO (Table 3). However, the pseudo-second order model best fits the experimental data regardless of the adsorbent, as shown in Figure S5 for the adsorption of MB by Clay_MA_, as an example.

**Figure 5:**
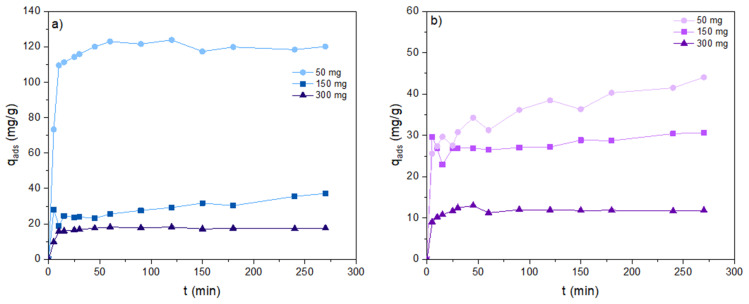
Uptake (q_ads_, mg/g) of MB (**a**) and CV (**b**) in the presence of AGO-1 for different masses of adsorbent.

For both dyes, the adsorption process was studied using different masses of adsorbents (from 50 to 300 mg) and the obtained results are shown for AGO-1 (Figure 5) and for Clay_MA_ and pyrophyllite (Figure S6), in terms of q_ads_ vs. contact time. Remarkably, higher uptakes were obtained with a lower mass of AGO-1 for both dyes. For the raw materials from Morocco, to obtain higher uptakes, it was necessary to increase the mass of adsorbent, as presented for phyrophyllite and Clay_MA_ with both dyes in Figure S6.

Noteworthy, in the case of AGO-1, higher q_ads_ values were observed when using the lowest mass of adsorbent (50 mg), with maximum values of ca. 120 and 45 mg/g for MB and CV, respectively (Figure 5).

In the case of the raw materials, for MB dye, higher amounts (150 or even 300 mg) are necessary for achieving much lower values of maximum uptake (ca. 40 and 25 mg/g for Clay_MA_ and Pyr, respectively (Figure S4)). In the case of CV dye, only Clay_MA_ shows a higher uptake with lower amounts, ca. 34 and 8 mg/g for Clay_MA_ and Pyr, respectively (Figure S4). These findings support the utilization of AMD precipitates as adsorbents in water treatments.

The adsorption isotherms were evaluated using the most commonly used Freundlich and Langmuir models. The Freundlich model assumes that adsorption takes place at sites with different energies on heterogeneous surfaces and that multilayer adsorption occurs; it is expressed in linear form by Equation (3):

(3) $$\ln\left(q_{e}\right)=\ln\left(K_{F}\right)+\frac{1}{n}\ln\left(C_{e}\right)$$

where *K_F_* and 1/*n* are the Freundlich constants [28].

The Langmuir isotherm assumes that adsorption occurs on a finite number of energetically identical sites until a monolayer is formed [28]. The linear form of the Langmuir equation is presented in Equation (4):

(4) $$\frac{C_{e}}{q_{e}}=\frac{1}{K_{L}q_{m}}+\frac{1}{q_{m}}C_{e}$$

where *K_L_* is the Langmuir constant (L/mg) and q_m_ is the maximum adsorption capacity.

**Figure 6:**
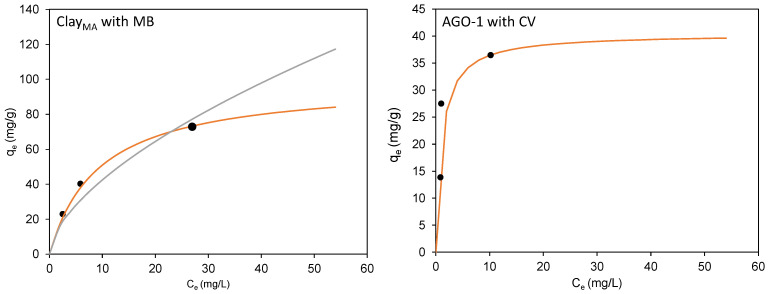
Langmuir (**−**) and Freundlich (**−**) isotherms for the adsorption of MB on Clay_MA_ and of CV on AGO-1. Symbols (●) represent the experimental data.

The isotherm model that best fits the experimental data is chosen based on the value of the correlation coefficient (R^2^) (Table S8). The fitting results are shown in Figure 6 for the adsorption of MB on Clay_MA_ and of CV on AGO-1, which were chosen as examples.

For both the dyes, in the case of AGO-1, the best fitting of the experimental adsorption data was obtained with the Langmuir equation, as shown by the better values of R^2^ (0.9945 for MB and 0.9864 for CV) compared to those of the Freundlich model (0.9461 for MB and 0.9026 for CV), which are, however, rather high. This result suggests that interpreting the adsorption phenomenon with the Freundlich model might be reasonable, since AGO-1 is composed of different materials (soil and plants), which makes the adsorption sites energetically heterogeneous. Further experimental data are needed to prove this hypothesis. The Langmuir isotherm also better describes the adsorption data in the case of the two raw adsorbents, although for the adsorption of CV on pyrophillite, the values of R^2^ are practically the same for the two models. These results indicate that homogeneous adsorption sites with similar affinities are present on the surface of the adsorbents. Table 4 shows the parameters of the Langmuir isotherm for the adsorption of MB and CV on Clay_MA_, Pyr, and AGO-1.

Interestingly, for Clay_MA_, the maximum adsorbed amount is similar for both the dyes (q_m_ = ca. 18 mg/g), suggesting that differences in the structure of the molecules in terms of steric hindrance do not influence the adsorption capacity. Conversely, in the case of pyrophillite, the best results are obtained with the smaller MB molecule, for which the q_m_ value is 30.8 mg/g. The differences observed in the adsorption capacities with Clay_MA_ and Pyr could be related to the agglomeration of the clay particles, which leads to a decrease in the surface area available for adsorption [29]. Unlike what is observed with raw adsorbents, AGO-1 shows the highest performance for CV adsorption (q_m_ = 40.5 mg/g), while the maximum adsorbed amount of MB is similar to that of Clay_MA_ (q_m_ = 16.0 mg/g).

**Figure 7:**
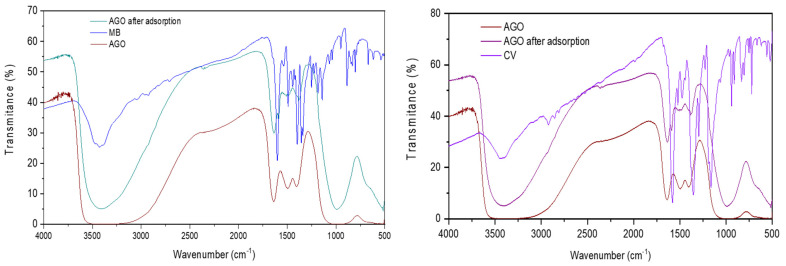
FTIR spectra of the dyes, AGO before and after adsorption of dyes.

FTIR analysis was used to study the stability of the AMD precipitates. Figure 7 shows the FTIR spectra of MB and CV before and after AGO adsorption.

All FTIR spectra display the characteristic bands of the dyes and the typical bands of the adsorbents before the adsorption process. After adsorption, some bands of the dyes were identified in the adsorbent spectrum, confirming the adsorption of the dye by AGO. Additionally, the absorbance bands of AGO remained in the same position before and after adsorption, indicating the stability of the material throughout the process.

**Scheme 1:**
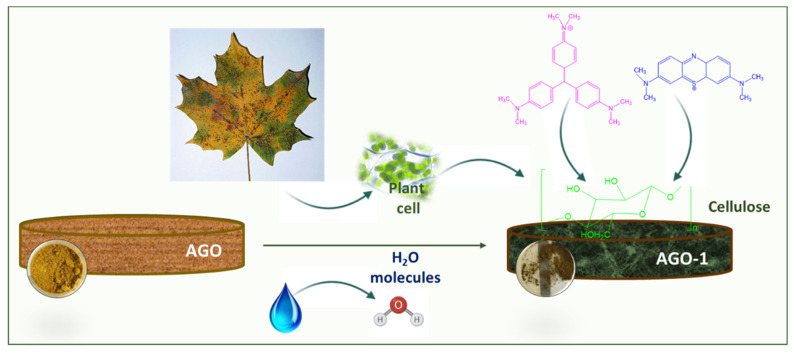
Adsorption process of the AGO-1 to remove the dyes.

The results obtained for AGO-1 demonstrate that materials from AMD precipitates are potentially efficient adsorbents for removing organic pollutants from water. The presence of organic matter in AGO-1 alongside the inorganic AGO improves its adsorption capacities, due to the presence of cellulose molecules (Scheme 1).

As mentioned before, few works are reported in the literature using AMD precipitates for the adsorption of inorganic and organic pollutants. For example, AMD precipitates proved to also be excellent adsorbents for metal ions, as noted in the work of [10], where they were found to adsorb Fe^3+^, Zn^2+^, Cu^2+^, and Mn^2+^ with uptakes from 84 to 96%. Therefore, our work contributes to the use of AMD precipitates for adsorbing organic pollutants in water, demonstrating their potential as a cost-effective solution for mitigating waste associated with acid mine drainage pollution.

## 3. Materials and Methods

### 3.1. Materials

Several raw materials from two different countries (Portugal and Morocco) were used as adsorbents. From the North of Portugal, iron-rich AMD precipitates (with the references AGO, AGO-1, CF, and V9) were compared with raw clay from the Middle Atlas region (Clay_MA_) [22], and a sample composed of the mineral pyrophyllite (Pyr) came from the Southern region of Morocco. The location and source nature of the materials are presented in Figure S1. Regarding AMD precipitates, the sample CF was obtained at the end of the limestone channel (neutralization stage) of the passive water treatment system of the Jales Mine (District of Vila Real, Pt, 41°46′ N, 7°59′ W). AGO and AGO-1 samples are also products of the passive water treatment process from the Argozelo Mine (District of Bragança, Pt, 41°39′ N 6°36′ W). AGO-1 differs from AGO by the presence of filamentous algae, which provides the sample with organic matter. Finally, V9 came from the streambed of a typical AMD-affected stream. It is an iron-rich AMD precipitate obtained by the raw natural attenuation processes of AMD from the Valdarcas Mine (District of Viana do Castelo, Pt, 41°52′9″ N, 8°42′19″ W). Prior to use, AMD precipitates were dried at low temperatures to avoid mineralogical transformations (40 °C during 24 h). The samples from Morocco were instead first sieved to a 63 μm fraction and then dried at 105 °C for 24 h. Methylene blue (MB, methylthioninium chloride, C_16_H_18_ClN_3_S) and crystal violet (CV, tris(4-(dimethylamino)phenyl)methylium chloride, C_25_N_3_H_30_Cl) were purchased from Merck (Figure S2). The dye solutions were prepared with deionized water produced with an ultrapure water system (Milli-Q, EQ 7000).

### 3.2. Adsorption Experiments

Adsorption experiments were performed in a semi-batch reactor containing a suitable mass of adsorbent and 250 mL of dye solution at the desired concentration. The suspension was stirred (100 rpm) at room temperature until adsorption equilibrium was reached. Periodically, sample aliquots were withdrawn from the reactor, centrifuged at 6000 rpm for 20 min, and filtered. The filtrate was analyzed by UV-vis spectroscopy at λmax = 664 nm and 591 nm, the characteristic wavelengths of MB and CV, respectively, using a UV-vis Spectrophotometer UV-2501PC from Shimadzu. The values of the dye concentration in the solution were obtained by interpolation of the absorbance values at different times in the calibration curves. All the results were conducted in duplicate, with the average deviation between the experimental and predicted results within ±5%. The removal percentage (R, %) of the dye was calculated from the initial concentration (C_0_, ppm) and the concentration at time *t* (C_t_, ppm) by Equation (5):

(5) $$R\left(\%\right)=\frac{\left(C_{0}-C_{t}\right)}{C_{0}}\times 100$$

The mass balance (Equation (6)) calculated the average amount of dye adsorbed, per unit of adsorbent mass at time *t*, q_ads_ (mg/g):

(6) $$q_{ads}\left(\mathrm{mg}/g\right)=\frac{(C_{0}-C_{t})\times V}{m}$$

where *m* (g) is the mass of dry adsorbent and *V* (L) is the volume of dye solution.

### 3.3. Experimental Design

The optimization of the adsorption process was followed by a response surface methodology (RSM) with a Box–Behnken Design (BBD) of three independent factors, namely, adsorbents (X_1_), mass (mg, X_2_), and dye concentration (ppm, X_3_), each on three levels with equidistant values (−1, 0, and +1), with −1 and +1 being the lowest and the highest level, respectively. For X_1_, a previous screening with AGO, pyrophyllite, Clay_MA_, and CF was carried out using 25 mL of an MB solution (30 ppm), and the first three samples were selected for the experimental design. Table S2 presents the independent process variables with their lowest and highest values used to identify the experimental sets by BBD.

Central point conditions (X_2_ = pyrophyllite, X_2_ = 15 mg, and X_3_ = 17.5 ppm of each dye) were run in triplicate for the BBD analysis. The three selected factors were used to determine the model coefficients in quadratic terms by the predicted response in terms of removal efficiency or adsorption capacity (Y_m_), which describes the correlation between independent process variables and their interaction terms and is calculated by Equation (7):

(7) $$Y_{m}=\beta_{0}+\sum_{i=1}^{k}\beta_{i}X_{i}+\sum_{i=1}^{k-1}\sum_{j=2}^{k}\beta_{ij}{X_{i}X}_{j}+\sum_{i=1}^{k}\beta_{ii}X_{i}^{2}+\varepsilon$$

where $\beta_{0}$ is the intercept coefficient; $\beta_{i},\;\beta_{ii},\;\mathrm{and}\beta_{ij}$ (with *i* = 1, 2, 3; *j* = 1, 2, 3) are the linear coefficients, squared coefficients, and interaction coefficients, respectively. ${X_{i}\;\mathrm{and}\;X}_{j}$ are the coded independent variables and ε is the random error [20,21,22]. Model analysis, response surface generation, and ANOVA statistics were completed using Design-expert 12 software.

### 3.4. Adsorption Kinetic and Equilibrium Studies

For the adsorption kinetics studies, the adsorbents selected through the BBD model (AGO-1, Pyr, and ClayMA) were tested in a semi-batch reactor using the optimized conditions, i.e., 150 mg of adsorbent and 250 mL of dye solution with a concentration of 30 mg/L for MB and 17.5 mg/L for CV. The three adsorbents were also used for the adsorption equilibrium studies, performed at room temperature with the same solution volume and dye concentration but using an initial mass of solid ranging from 50 to 300 mg.

### 3.5. Characterization

Several characterization techniques were used, such as X-ray diffraction (XRD), Scanning Electron Microscopy (SEM) with Energy Dispersive X-ray Spectroscopy (EDX), N_2_ adsorption/desorption analysis, and Fourier Transform Infrared Spectroscopy (FTIR).

The XRD patterns for mineralogical identifications were obtained by powder diffraction with a Philips PW1710 diffractometer using Cu-Kα radiation (40 kV, 30 mA), equipped with an automatic divergence slit and graphite monochromator. The clay sample from Morocco was analyzed in a fraction < 2 µm after chemical and thermal treatments [22].

SEM-EDX analysis was performed with a Phenom ProX microscope equipped with an EDX detector (Phenom-World BV, Amsterdam, The Netherlands). All results were examined using the ProSuite (3.0.1.6) software integrated with the Phenom Element Identification (3.8.4.0) software, which allowed the quantification of the elements expressed in either weight or atomic concentration. For the analysis, samples were added to an aluminum pin stub with electrically conductive carbon adhesive tape (PELCO Tabs™) and were imaged without coating. The aluminum pin stub was then placed inside a Phenom Charge Reduction Sample Holder (CHR) and different points were analyzed for elemental composition. EDS analysis was conducted at 15 kV with an intensity map.

Textural characterization was based on the N_2_ adsorption isotherms, determined at −196 °C with Nova 4200e (Quantachrome Instruments, Boynton Beach, FL, USA) equipment. Before analysis, the samples were heated for 3 h under vacuum up to 150 °C (heating rate, 5 °C min^−1^). Surface areas were assessed by applying the BET equation, whereas the micropore volume (V_micro_) and mesopore surface area (S_meso_) were calculated using the *t*-plot method.

FTIR measurements were carried out at room temperature using a PerkinElmer Spectrum Two spectrometer, equipped with an ATR accessory. A diamond prism was used as the waveguide. All spectra were recorded with a resolution of 4 cm^−1^ in the wavelength region 4000–400 cm^−1^ by averaging 16 scans.

## 4. Conclusions

The current study reveals that AMD precipitates can be cost-effective and easily accessible alternative adsorbents for effectively removing organic pollutants in wastewater treatments. Various adsorbents derived from AMD wastes in Portugal (AGO, AGO-1, CF, and V9) were compared with two raw materials from Morocco (Clay_MA_ and pyrophyllite) for their ability to remove dyes from water treatment. The Langmuir isotherm described the adsorption equilibrium of the three selected adsorbents, while the adsorption kinetics followed the pseudo-second order model. Although the raw materials demonstrated a favorable adsorption performance, AMD precipitates exhibited superior removal capacities, achieving the highest q_m_ value for crystal violet (40.5 mg/g) and a q_m_ (16.0 mg/g) like that of the raw clay for methylene blue. The adsorption performance of AGO-1 is attributed to the presence of cellulose molecules, which enhance the adsorption process. The Langmuir isotherm described the adsorption equilibrium of the best adsorbents, while the adsorption kinetics followed the pseudo-second order model. Hence, it can be inferred that utilizing readily available and recycled materials in the adsorption process is efficient, resulting in reduced process costs and improved sustainability in the context of the circular economy.

## Figures and Tables

**Table 1:** Chemical analysis of the samples.

Element (wt%)	AGO ^1^	AGO-1 ^1^	V9 ^1^	CF ^1^	ClayMA ^2^	ClayMA ^1^	Pyr ^1^
C	76.32	46.81	5.60	37.34	nd	nd	nd
O	17.51	40.71	37.80	42.79	nd	55.70	61.05
Si	0.96	1.43	0.20	0.71	27.06	21.18	20.69
Fe	4.32	6.57	53.0	17.59	5.40	5.47	nd
Al	0.33	0.44	nd	0.13	12.05	10.96	14.07
Mg	nd	0.63	nd	1.44	1.86	1.11	0.25
Na	0.56	1.23	nd	nd	0.25	0.24	0.64
Ca	nd	0.31	nd	0.13	3.69	1.06	nd
K	nd	nd	nd	nd	3.68	1.98	1.40
Ti	nd	nd	nd	nd	0.51	nd	nd
S	nd	nd	nd	2.80	nd	nd	1.91

^1^ Chemical analysis performed by EDX; ^2^ Chemical analysis performed by ICP-OES [22]; nd = not detected.

**Table 2:** Fit statistics results of both responses for the adsorption of MB and CV dyes.

	MB		CV	
	Y_1_	Y_2_	Y_1_	Y_2_
R^2^	0.9664	0.9844	0.9507	0.9810
Adjusted R^2^	0.9059	0.9564	0.8619	0.9467
Predicted R^2^	0.4636	0.7526	0.2125	0.6978
Adequate Precision	13.3306	19.2163	9.5912	18.5072
Standard Deviation	7.16	3.99	7.37	3.05
Mean	84.18	27.09	56.14	18.88
Coefficient of Variation (%)	8.51	14.73	13.13	16.15

**Table 3:** The optimal experimental conditions for the adsorption of MB and CV dyes in function of the uptake (Y_2_, mg/g).

	MB		CV	
Adsorbent (X_1_)	Clay_MA_		pyrophyllite	
m (mg, X_2_)	15		15	
[dye] (ppm, X_3_)	30		17.5	
	Predicted	Experimental	Predicted	Experimental
R (%)	82.42	89.90	82.45	83.10
q_ads_ (mg/g)	45.38	44.90	20.53	20.10

**Table 4:** Parameters of the Langmuir isotherm for the adsorption of MB and CV on ClayMA, pyrophillite, and AGO-1.

Dye	MB			CV		
Parameters	q_m_ (mg/g)	KL (L/mg)	R^2^	qm (mg/g)	KL (L/mg)	R^2^
Clay_MA_	17.7	0.60	0.9945	17.9	0.50	0.9468
Pyr	30.8	3.12	0.9939	25.4	2.04	0.9916
AGO-1	16.0	0.21	0.9945	40.5	0.91	0.9864

## Data Availability

Data are contained within the article and Supplementary Materials.

## Supplementary Materials

The following are available online at https://www.mdpi.com/article/10.3390/molecules29153521/s1, Figure S1: Map of Portugal and Morocco with the identification of the materials and the molecular structure of the MB and CV dyes. Figure S2: FTIR spectra of the AGO and AGO-1. Figure S3: XRD patterns of the AGO and AGO-1, respectively. Figure S4: 3D-RSM plots for MB adsorption, where the response is reported as a function of two of the independent variables, while keeping the third constant: Y1 (a, c, e) and Y2 (b, d, f). Figure S5: Fitting of experimental data (qads vs. t) for MB adsorption on ClayMA using the PFO and PSO models. Symbols (●) represent the experimental data. Figure S6: Uptake (q_ads_, mg/g) of MB (a, c) and CV (b, d) in the presence of Pyrophyllite and ClayMA for different mass of adsorbent. Table S1: Textural properties of the samples. Table S2: Independent factors and levels used for Box-Behnken design. Table S3: BBD for the three independent variables for responses Y1 and Y2 of MB and CV adsorption process. Table S4: Analysis of variance (ANOVA) for the response Y1, removal efficiency (%) of MB and CV dyes in adsorption process. Table S5: ANOVA for the response Y2, quantity adsorbed (q_ads_, mg/g) of MB and CV dyes in adsorption process. Table S6: Second-order polynomial equations for the MB and CV adsorption processes as a function of Y1 (R (%)) and Y2 (q_ads_ (mg/g)). Table S7: The parameters of the pseudo-first-order and pseudo-second-order models. Table S8: Freundlich and Langmuir equations for the adsorption of MB and CV on Clay_MA_, Pyrophillite, and AGO-1.
