# Supplementary material for: Acid Mine Drainage Precipitates from Mining Effluents as Adsorbents of Organic Pollutants for Water Treatment
Source: Molecules. 2024 Jul 26;29(15):3521. doi: 10.3390/molecules29153521 (PMC11314495; doi:10.3390/molecules29153521)
Supplement: Supplementary file 1 [file molecules-29-03521-s001.zip › molecules-3082777-supplementary.pdf]

Supplementary Information

Acid mine drainage-precipitates from mining effluents as adsorbents of organic pollutants for water treatment

Marta S.F. Oliveira [1], Ouissal Assila [1], António M. Fonseca [1,2], Pier Parpot [1,2], Teresa Valente [3], Elisabetta Rombi [4], Isabel C. Neves [1,2*]

1 CQUM, Centre of Chemistry, Chemistry Department, University of Minho, Campus de Gualtar, 4710-057, Braga, Portugal; martaoliveira@quimica.uminho.pt (M.S.F.O.); ouissal.assila@usmba.ac.ma (O.A.); amcf@quimica.uminho.pt (A.M.F.); parpot@quimica.uminho.pt (P.P.)
2 CEB - Centre of Biological Engineering, University of Minho, Campus de Gualtar, 4710-057 Braga, Portugal;
3 ICT, Institute of Earth Sciences, Pole of the University of Minho, 4710-057 Braga, Portugal; teresav@dct.uminho.pt (T.V.)
4 Department of Chemical and Geological Sciences, University of Cagliari, Complesso Universitario di Monserrato, 09042 Monserrato, Italy; rombi@unica.it (E.R.)
* Correspondence: authors: ineves@quimica.uminho.pt (I.C.N.)

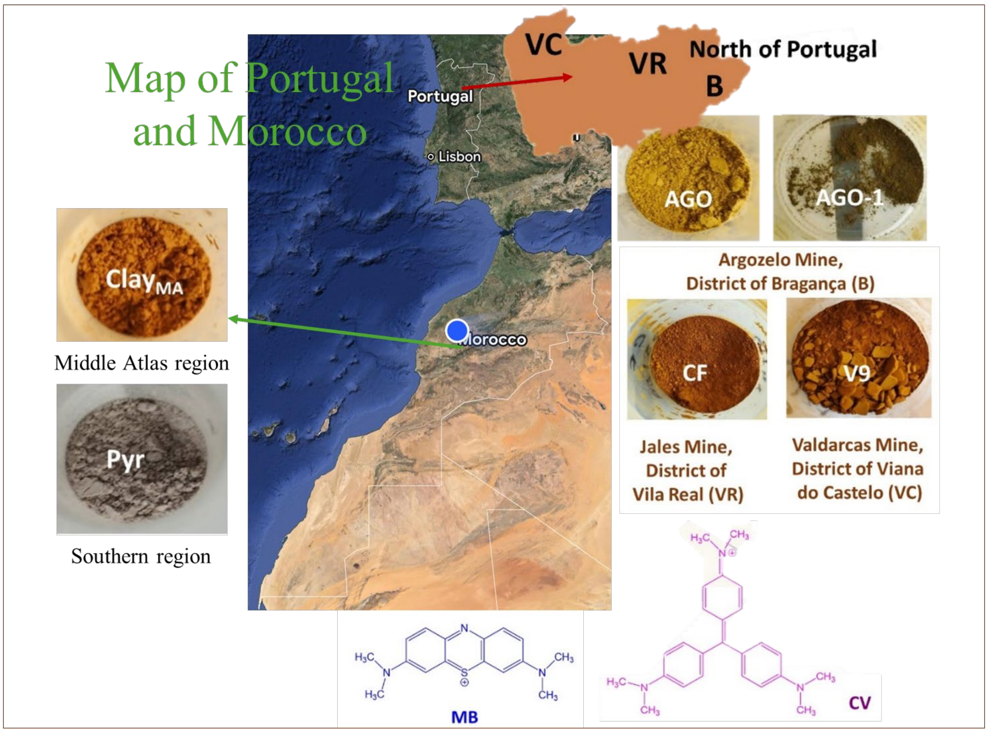

Figure S1: Map of Portugal and Morocco with the identification of the materials and the molecular structure of the MB and CV dyes.

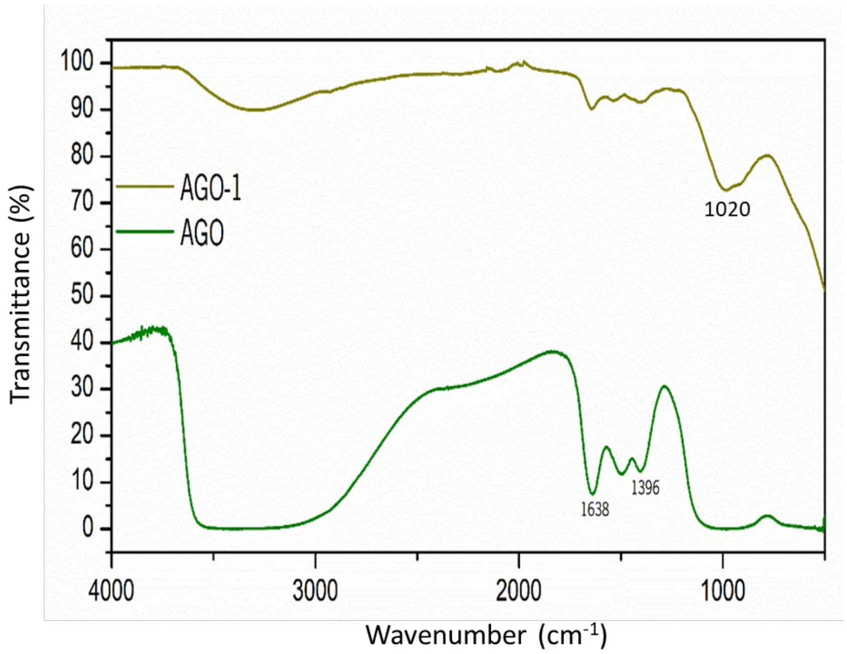

Figure S2: FTIR spectra of the AGO and AGO-1.

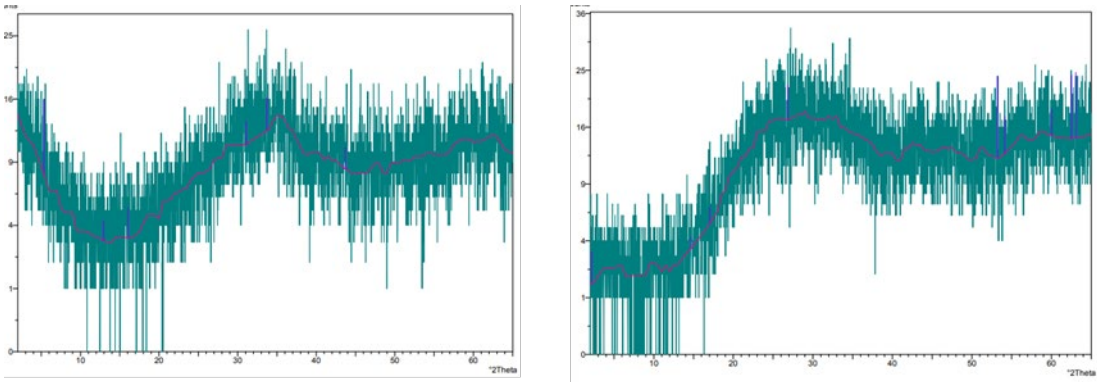

Figure S3: XRD patterns of the AGO and AGO-1, respectively.

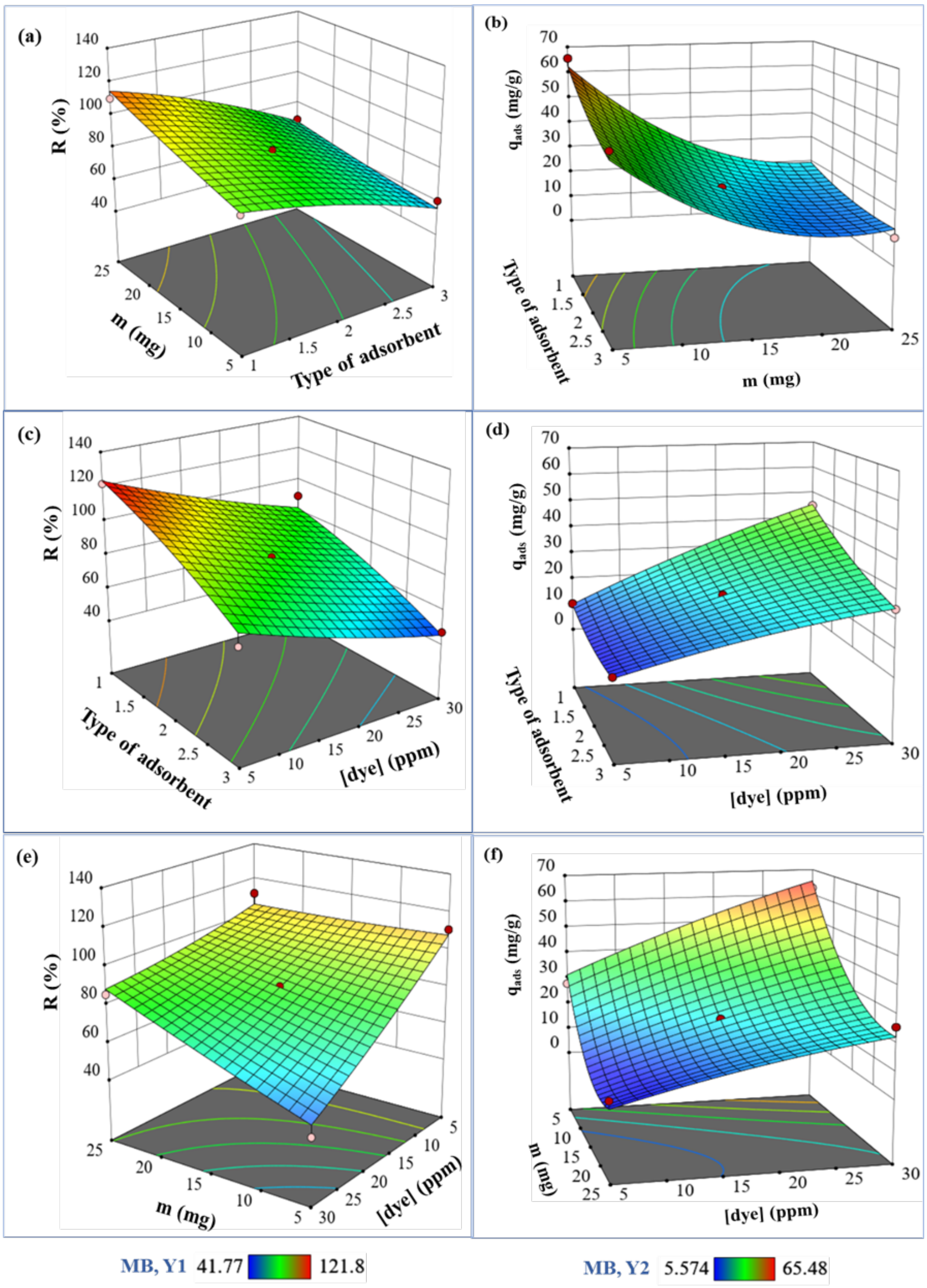

Figure S4: 3D-RSM plots for MB adsorption, where the response is reported as a function of two of the independent variables, while keeping the third constant: Y1 (a, c, e) and Y2 (b,d,f).

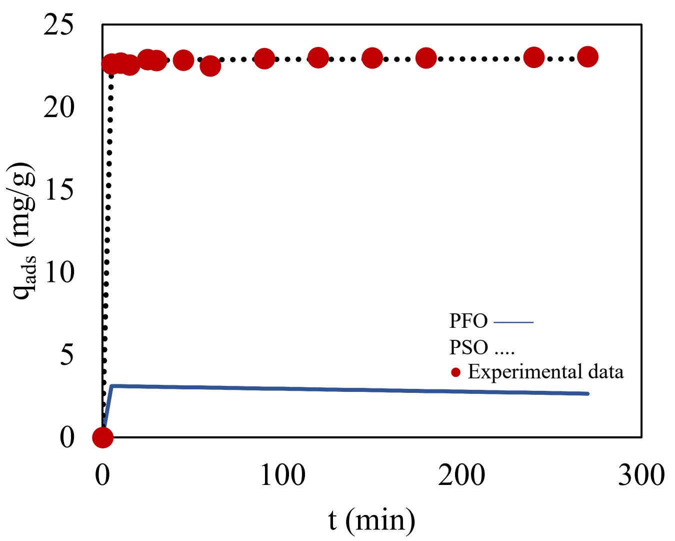

Figure S5: Fitting of experimental data (q_{ads} *vs*. t) for MB adsorption on $Clay_{MA}$ using the PFO and PSO models. Symbols (●) represent the experimental data.

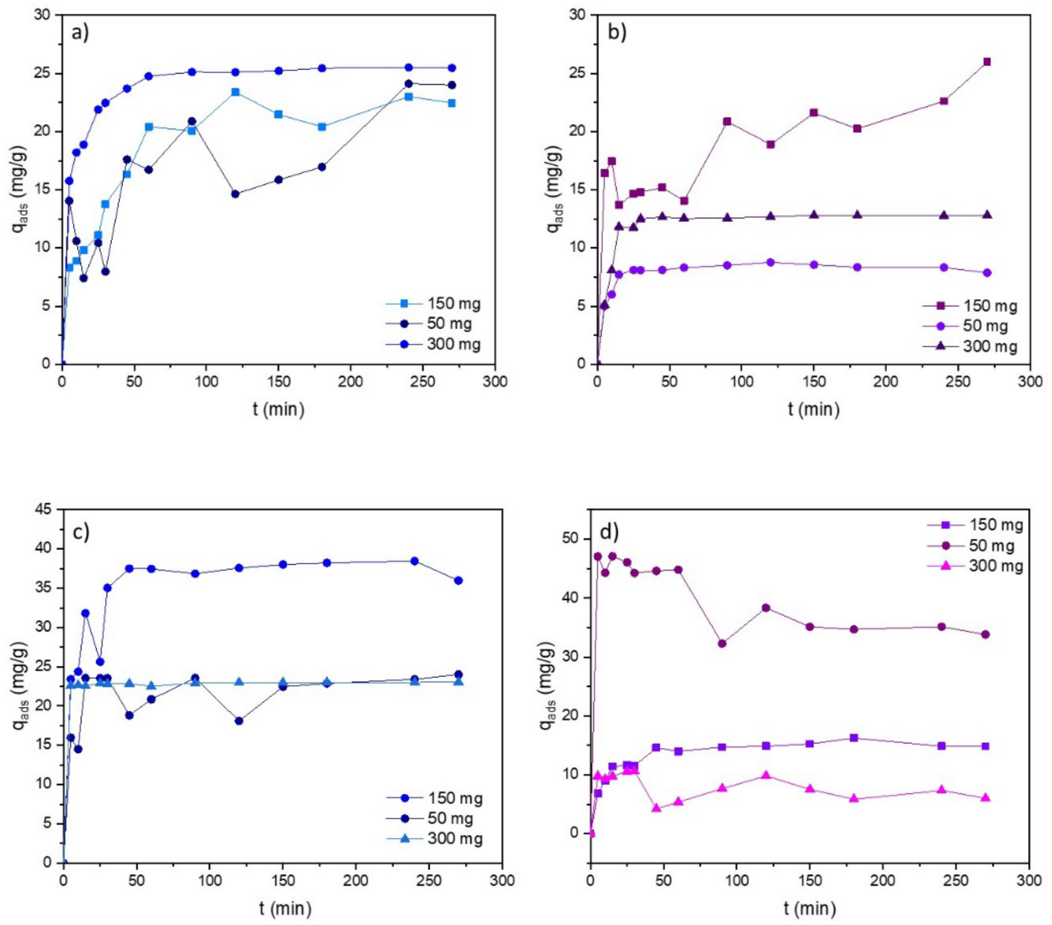

Figure S6: Uptake (q_{ads}, mg/g) of MB (a, c) and CV (b, d) in the presence of Pyrophyllite and $Clay_{MA}$ for different mass of adsorbent.

Table S1: Textural properties of the samples.

Sample	S_{BET} (m²/g)[a]	V_p (cm³/g)[b]
AGO	79	0.124
AGO-1	369	0.489
$Clay_{MA}$	23	0.039
Pyrophyllite	12	0.030

[a]determined by the BET method; [b]calculated at p/p_0 = 0.95.

Table S2: Independent factors and levels used for Box-Behnken design.

Coded factor	Factor	Coded level		
		-1	0	+1
X_1	Type of adsorbent	$Clay_{MA}$ (1)	Pyrophyllite (2)	AGO (3)
X_2	m (mg)	5	15	25
X_3	[dye] (ppm)	5	17.5	30

Table S3: BBD for the three independent variables for responses Y1 and Y2 of MB and CV adsorption process.

Code factor	X_1	X_2	X_3	(methylene blue) MB		(crystal violet) CV	
				Y1	Y2	Y1	Y2
Assays	Type of adsorbent	m (mg)	[dye] (ppm)	R (%)	q_{ads} (mg/g)	R (%)	q_{ads} (mg/g)
1	1	5	17.5	87.3	65.48	46.63	34.98
2	3	5	17.5	60.71	45.53	44.76	33.57
3	1	25	17.5	110.05	16.51	82.18	12.33
4	3	25	17.5	69.16	10.37	41.11	6.17
5	1	15	5.0	121.80	10.15	45.24	3.77
6	3	15	5.0	73.79	6.15	31.75	2.64
7	1	15	30.0	89.89	44.94	63.89	31.94
8	3	15	30.0	47.13	23.57	32.44	16.22
9	2	5	5.0	110.79	27.70	44.23	11.06
10	2	25	5.0	111.48	5.574	69.46	3.47
11	2	5	30.0	41.77	62.66	31.29	46.94
12	2	25	30.0	85.42	25.63	61.80	18.54
13	2	15	17.5	84.38	21.10	82.28	20.57
14	2	15	17.5	85.08	20.06	83.08	20.07
15	2	15	17.5	84.00	21.00	82.00	20.97

Table S4: Analysis of variance (ANOVA) for the response Y1, removal efficiency (%) of MB and CV dyes in adsorption process

dye	Source	Sum of Squares	df	Mean Square	F-value	p-value
**MB**	Model	7381.76	9	820.20	15.98	0.0035
	X_1- Type of adsorbent	3130.38	1	3130.38	61.00	0.0006
	X_2- m (mg)	713.29	1	713.29	13.90	0.0136
	X_3- [dye] (ppm)	2951.04	1	2951.04	57.50	0.0006
	X_1X_2	51.12	1	51.12	0.9961	0.3641
	X_1X_3	6.89	1	6.89	0.1343	0.7290
	X_2X_3	461.39	1	461.39	8.99	0.0302
	X_1^2	43.87	1	43.87	0.8549	0.3976
	X_2^2	2.16	1	2.16	0.0421	0.8454
	X_3^2	16.48	1	16.48	0.3212	0.5954
	Residual	256.61	5	51.32		
	Lack of Fit	256.01	3	85.34	284.33	0.0035
	Pure Error	0.6003	2	0.3001		
	Cor Total	7638.37	14			
**CV**	Model	5234.46	9	581.61	10.71	0.0089
	X_1- Type of adsorbent	965.34	1	965.34	17.77	0.0084
	X_2- m (mg)	960.05	1	960.05	17.68	0.0085
	X_3- [dye] (ppm)	0.1994	1	0.1994	0.0037	0.9540
	X_1X_2	384.20	1	384.20	7.07	0.0449
	X_1X_3	80.61	1	80.61	1.48	0.2774
	X_2X_3	6.97	1	6.97	0.1283	0.7348
	X_1^2	1273.93	1	1273.93	23.46	0.0047
	X_2^2	384.83	1	384.83	7.09	0.0448
	X_3^2	1559.16	1	1559.16	28.71	0.0030
	Residual	271.55	5	54.31		
	Lack of Fit	270.92	3	90.31	287.48	0.0035
	Pure Error	0.6283	2	0.3141		
	Cor Total	5506.01	14			

Table S5: ANOVA for the response Y2, quantity adsorbed (q_{ads}, mg/g) of MB and CV dyes in adsorption process.

dye	Source	Sum of Squares	df	Mean Square	F-value	p-value
**MB**	Model	5035.35	9	559.48	35.12	0.0005
	X_1- Type of adsorbent	330.99	1	330.99	20.77	0.0061
	X_2- m (mg)	2566.36	1	2566.36	161.08	< 0.0001
	X_3- [dye] (ppm)	1437.18	1	1437.18	90.20	0.0002
	X_1X_2	47.68	1	47.68	2.99	0.1442
	X_1X_3	75.50	1	75.50	4.74	0.0814
	X_2X_3	55.58	1	55.58	3.49	0.1208
	X_1^2	19.26	1	19.26	1.21	0.3217
	X_2^2	485.86	1	485.86	30.49	0.0027
	X_3^2	11.97	1	11.97	0.7511	0.4257
	Residual	79.66	5	15.93		
	Lack of Fit	79.01	3	26.34	80.37	0.0123
	Pure Error	0.6554	2	0.3277		
	Cor Total	5115.01	14			
**CV**	Model	2397.25	9	266.36	28.64	0.0009
	X_1- Type of adsorbent	74.49	1	74.49	8.01	0.0367
	X_2- m (mg)	925.30	1	925.30	99.48	0.0002
	X_3- [dye] (ppm)	1074.18	1	1074.18	115.48	0.0001
	X_1X_2	5.66	1	5.66	0.6090	0.4705
	X_1X_3	53.22	1	53.22	5.72	0.0622
	X_2X_3	108.27	1	108.27	11.64	0.0190
	X_1^2	24.33	1	24.33	2.62	0.1667
	X_2^2	53.06	1	53.06	5.70	0.0625
	X_3^2	69.09	1	69.09	7.43	0.0415
	Residual	46.51	5	9.30		
	Lack of Fit	46.10	3	15.37	75.58	0.0131
	Pure Error	0.4067	2	0.2033		
	Cor Total	2443.76	14			

Table S6: Second-order polynomial equations for the MB and CV adsorption processes as a function of Y1 (R (%)) and Y2 (q_{ads} (mg/g)).

Dye	Equations
MB	Y1= 84.49 - 19.78X_1 + 9.44X_2 - 19.21X_3 - 3.57X_1X_2 + 1.31X_1X_3 + 10.74X_2X_3 - 3.45X_1^2 + 0.7654X_2^2 + 2.11X_3^2 (Eq. 1)<br><br>Y2= 20.72 - 6.43X_1 - 17.91X_2 +13.4X_3 + 3.45X_1X_2 - 4.34X_1X_3 - 3.73X_2X_3 + 2.28X_1^2 + 11.47X_2^2 - 1.8X_3^2 (Eq. 2)
CV	Y1= 82.45 - 10.98X_1 + 10.95X_2 - 0.1579X_3 - 9.8X_1X_2 - 4.49X_1X_3 + 1.32X_2X_3 - 18.57X_1^2 - 10.21X_2^2 - 20.55X_3^2 (Eq. 3)<br><br>Y2= 20.54 - 3.05X_1 - 10.75X_2 +11.59X_3 - 1.19X_1X_2 - 3.65X_1X_3 - 5.2X_2X_3 - 2.57X_1^2 + 3.79X_2^2 - 4.33X_3^2 (Eq. 4)

Table S7: The parameters of the pseudo-first-order and pseudo-second-order models.

	$Clay_{MA}$			
	MB		CV	
Kinetic model	PFO	PSO	PFO	PSO
Equation	y = -0.061x+3.258	y = 0.025x+0.149	y = -0.065x+2.538	y = 0.063x+0.475
R^2	0.6727	0.9248	0.8373	0.9855
	**Pyrophyllite**			
	PFO	PSO	PFO	PSO
Equation	y = 0.015x+1.174	y = 0.036x+1.101	y = 0.009x+2.113	y = 0.047x+0.686
R^2	0.5516	0.9601	0.2589	0.9480
	**AGO-1**			
	PFO	PSO	PFO	PSO
Equation	y = -0.010x+2.180	y = 0.035x+0.186	y = -0.011x+1.693	y = 0.036x 0.055
R^2	0.4088	0.9916	0.2743	0.9987

Table S8: Freundlich and Langmuir equations for the adsorption of MB and CV on $Clay_{MA}$, Pyrophillite, and AGO-1.

	**$Clay_{MA}$**	
	MB	CV

Isotherm model	Freundlich	Langmuir	Freundlich	Langmuir
Equation	y = -0.588x+4.725	y = 0.047x-0.126	y = 0.299x-2.025	y = 0.056x+0.114
R^2	0.9265	0.9945	0.6948	0.9468
	**Pyrophyllite**			
	Freundlich	Langmuir	Freundlich	Langmuir
Equation	y = -0.403x+3.511	y = 0.033x+0.010	y = 0.182x+2.808	y = 0.039x+0.019
R^2	0.1487	0.9939	0.9928	0.9916
	**AGO-1**			
	Freundlich	Langmuir	Freundlich	Langmuir
Equation	y = -1.076x+6.371	y = 0.063x-0.293	y = 0.728x-2.979	y = 0.025x+0.027
R^2	0.9461	0.9945	0.9026	0.9864
